# Addressing broader reproductive health needs of female sex workers through integrated family planning/ HIV prevention services: A non-randomized trial of a health-services intervention designed to improve uptake of family planning services in Kenya

**DOI:** 10.1371/journal.pone.0219813

**Published:** 2019-07-24

**Authors:** Lisa Dulli, Samuel Field, Rose Masaba, John Ndiritu

**Affiliations:** 1 Family Health International (FHI 360), Durham, NC, United States of America; 2 Elizabeth Glaser Pediatrics AIDS Foundation, Nairobi, Kenya; 3 FHI 360, Juba, Nabari, South Sudan; Centre for the AIDS Programme of Research in South Africa (CAPRISA), SOUTH AFRICA

## Abstract

**Background:**

Despite considerable efforts to prevent HIV and other sexually transmitted infections (STI) among female sex workers (FSW), other sexual and reproductive health (SRH) needs, such preventing unintended pregnancies, among FSW have received far less attention. Programs targeting FSW with comprehensive, accessible services are needed to address their broader SRH needs. This study tested the effectiveness of an intervention to increase dual contraceptive method use to prevent STIs, HIV and unintended pregnancy among FSW attending services in drop-in centers (DIC) in two cities in Kenya. The intervention included enhanced peer education, and routine screening for family planning (FP) needs plus expanded non-condom FP method availability in the DIC.

**Methods:**

We conducted a two-group, pre-/posttest, quasi-experimental study with 719 FSW (360 intervention group, 359 comparison group). Participants were interviewed at baseline and 6 months later to examine changes in condom and non-condom FP method use.

**Results:**

The intervention had a significant positive effect on non-condom, FP method use (OR = 1.38, 95%CI (1.04, 1.83)), but no effect on dual method use. Consistent condom use was reported to be high; however, many women also reported negotiating condom use with both paying and non-paying partners as difficult or very difficult. The strongest predictor of consistent condom use was partner type (paying versus non-paying/emotional); FSW reported both paying and non-paying partners also influence non-condom contraceptive use. Substantial numbers of FSW also reported experiencing sexual violence by both paying and non-paying partners.

**Conclusions:**

Self-reported difficulties with consistent condom use and the sometimes dangerous conditions under which they work leave FSW vulnerable to unintended pregnancy STIs/HIV. Adding non-barrier FP methods to condoms is crucial to curb unintended pregnancies and their potential adverse health, social and economic consequences. Findings also highlight the need for additional strategies beyond condoms to reduce HIV and STI risk among FSW.

**Trial registration:**

Clinicaltrials.gov NCT01957813

## Introduction

Considerable efforts to prevent and treat human immunodeficiency virus (HIV) and other sexually transmitted infections (STI) have targeted female sex workers (FSW) over the past decade. Other sexual and reproductive health (SRH) needs of FSW have received far less attention [1]; yet, factors that increase risk of HIV and other STIs—multiple sexual partners, low/inconsistent use of barrier protection (e.g. condoms)–also increase other SRH health problems risk, such as unintended pregnancy; health risks from unsafe induced abortions are also an important issue among FSWs reported in many countries [2–8].

Condom use has been a major focus of HIV and STI prevention strategies targeting FSWs. Efforts to increase condom use among FSW have been relatively successful with many studies reporting high condom use rates [2, 4, 9–12]; however, despite high self-reported condom use, use is often inconsistent [13–17]. Some studies also indicate incongruities between self-reported condom use and actual behavior [5, 9, 11]; women who report frequent or consistent condom use, also often report difficulty negotiating condom use or being paid more to engage in sex without condoms [15, 17]. Furthermore, using condoms with paying partners does not always translate into using condoms with non-paying or emotional partners [13, 14].

Non-condom, modern contraceptive method use among FSW is often quite low [2, 8, 9, 12, 18, 19] and among FSW who do use such methods, inconsistent use and frequent method switching are often reported [5, 8]. Continued high rates of unintended pregnancy and induced abortion among FSW highlight the need to improve access to and uptake of non-condom contraceptive methods in addition to condoms [5, 20].

### Kenya

A study examining contraceptive need and use among FSW in Kenya reported high unmet SRH needs; 52% of women reported ever having an unintended pregnancy and 37% reported ever having an induced abortion [21]. Current contraceptive use was low with only 54% reported using a non-condom, modern method, with or without condoms. Although 90% of women reported condom use at last sex with a paying partner, 46% stated negotiating condom use with clients was "difficult" or "impossible”[21]. Focus group discussion (FGD) participants added that FSWs may forget to use condoms while drunk or high [21].

Given the elevated risks for unintended pregnancies and HIV/STIs, FSWs need comprehensive, accessible SRH preventive services. We set out to test an intervention to increase non-condom, modern method and dual method use among FSWs attending health services at drop-in centers (DIC) in two Kenyan cities.

## Methods

We conducted a two-group, pre-/posttest, quasi-experimental study to test the effectiveness of an integrated health services intervention. Baseline data were collected as participants enrolled from June 20^th^ to September 27^th^, 2013 and endline data were collected between January 23, 2014 to May 12, 2014. Neither study participants nor study staff were blinded to treatment assignment. The study was approved by FHI 360’s Protection of Human Subjects Committee (PHSC) on September 23, 2011 and the Kenya Medical Research Institute’s (KEMRI) Ethics Committee on December 7, 2011. The study was registered with www.Clinicaltrials.gov, and although the study was registered prior to participant enrolment, due to administrative issues with the record that required modification, the study record was not released to the public on clinicaltrials.gov until September 4, 2013. No additional trials of this intervention have been conducted.

### Setting and sample

We conducted the study in Naivasha (intervention site) and Nanyuki (comparison site), Kenya, where tourists, migrant workers and military personnel have attracted a high number of FSW. Each town had one DIC providing basic HIV prevention services and linkages to care for HIV-positive clients.

Self-identified FSW were recruited sequentially as they presented to DICs in both sites until the required sample size was achieved. Eligible participants included women 16–49 years old reporting receiving money or goods in exchange for sex in the prior 6 months as a source of livelihood. Peer educators and DIC staff informed FSW of the study, referring interested women to data collectors stationed at the DICs. Informed consent was obtained prior to interviews, which were conducted in a private setting within the DIC. Both PHSC and KEMRI granted a waiver a parental consent for participants ages 16–17, consistent with the Kenya Ministry of Health’s national guidance on conducting research on sexual and reproductive health among adolescents in Kenya [22]. Participants were reimbursed 300 Kenya Shillings (KES) (approximately US$3.53) at each time point for travel costs and time to participate in interviews.

Sample size was based on the ability to detect a 15 percentage point difference from baseline to endline in dual method use between groups with a two-sided test with alpha = 0.05 and 80% power, assuming baseline dual method prevalence was 38% [21]. The resulting sample size, 300 participants in each group, was increased by 20% to account for possible attrition, resulting in a total sample of 360 women per site or 720 total.

### Intervention description

The experimental intervention was designed through a participatory process with local service providers and FSW representatives. Formative research included FGDs with FSW, peer educators and health providers in the intervention area [23]. A working group comprised of members of the same groups informed intervention design based on formative results.

Standard services available at both study DICs, staffed by a counselor and a nurse, included routine HIV counseling and testing, STI screening and treatment, and peer education. DICs also delivered basic FP counseling, supplied condoms and oral contraceptive pills (OCP), and referred women to local health facilities for other methods. The peer education program encompassed twelve 45–60 minute sessions covering topics related to: HIV; correct and consistent condom use; FP; alcohol and drug abuse; and gender-based violence.

The experimental intervention included all standard services plus: 1) Enhanced peer educator training on FP methods to promote dual protection and dual method use; 2) Routine screening for FP needs and counseling on consistent condom and dual method use at each clinic visit; and 3) Expanded onsite FP method availability including injectable contraceptives (DMPA), intra-uterine contraceptive devices (IUD), and implants, free of charge to clients. The expanded FP services were delivered according to Kenyan Ministry of Health (MOH) guidelines, consistent with current WHO medical eligibility criteria. Clients were provided standard counseling on all contraceptive methods available to them, as well as benefits and drawbacks of each method, so that each client could make an informed choice with the health provider as to the best method for her. This informed choice counseling and method selection was reinforced with a job aid called “*The Balanced Counseling Strategy Plus*,*”* distributed by the Population Council, to support providers in helping women select the most appropriate method for their individual circumstances.[24]

### Measures and statistical approach

The primary study outcomes were non-condom modern contraceptive method use and dual method use. Non-condom method use was defined as self-reported current use of: OCP, DMPA, implants, IUD, female sterilization, Standard Days Method (SDM), or Lactational Amenorrhea Method (LAM). Emergency contraception was not considered in this study.

Dual method use was defined as current use of a non-condom, modern method plus consistent condom use with all partners for the prior 30 days. Consistent condom use—defined as use at last sex and always over the past 30 days with all sex partners—was determined by asking participants about their sexual activities with both paying and non-paying partners.

### Other variables

We also calculated unmet contraceptive need, defined as the proportion of non-pregnant women who did not want to be pregnant ever (again) or within the next two years who were not currently using a non-condom modern method use. Unmet contraceptive need was divided into unmet need to space pregnancies (want to be pregnant, but not for 2+ years) or to limit pregnancies (never want to be pregnant (again)). Women who reported using only condoms, even consistent condom use with all partner types, were counted as having unmet contraceptive need due to the frequent inconsistencies between self-reported and actual condom use reported in the literature. Partner type classified as non-paying (emotional) partner, paying partner or both. Emotional partners were those with whom FSW reported having a non-paying, ongoing relationship such as a husband or regular boyfriend.

### Statistical analyses

Differences between study groups on baseline characteristics were assessed using the Chi Square test of independence for categorical variables and the Student t-test independent samples difference in means for continuous variables. Generalized linear mixed models were used to test our study hypotheses. Random effects were included to account for repeated measures from study participants. Fixed effects included measurement occasion, treatment exposure, and a time-by-treatment exposure interaction. All models controlled for age, sex and partner type. Our primary interest was the difference in difference (DD) estimate of the intervention’s impact. For all statistical tests reported here, we adopted .05 type I error rate when reporting statistical significance.

DD estimates are robust against confounding by unobserved, time-stable variables, but remain vulnerable to time-varying confounders [25]. Exploratory analyses revealed changes in relationship status strongly predicted the outcome and intervention group participants were more likely to gain a non-paying partner between time points than comparison group participants. Thus, we created a change in relationship status variable by subtracting each participant’s non-paying partner variable measured at baseline from their endline measurement to adjust for potential confounding. This variable was entered as both a main effect and an interaction with time.

Because of substantial missing data due to loss to follow-up (LTF), multiple imputation was carried out using the MI procedure in SAS version 9.3[26] to generate 50 imputed dataset to test our study’s hypotheses. All analytic covariates used in the regression modeling (i.e.main effects, interaction terms, and dependent variables) in addition to related variables were used in the imputation. We also included a set of auxiliary variables to improve the precision of the imputed missing values. These variables included baseline values of: HIV status, sought HIV and/or STI service in past 6 months, desire for children/additional children, history of elective abortion, perceived susceptibility and severity of an unintended pregnancy, perceived barriers to FP service use, perceived benefits of FP use,.

## Results

### Descriptive analyses

**Fig 1:**
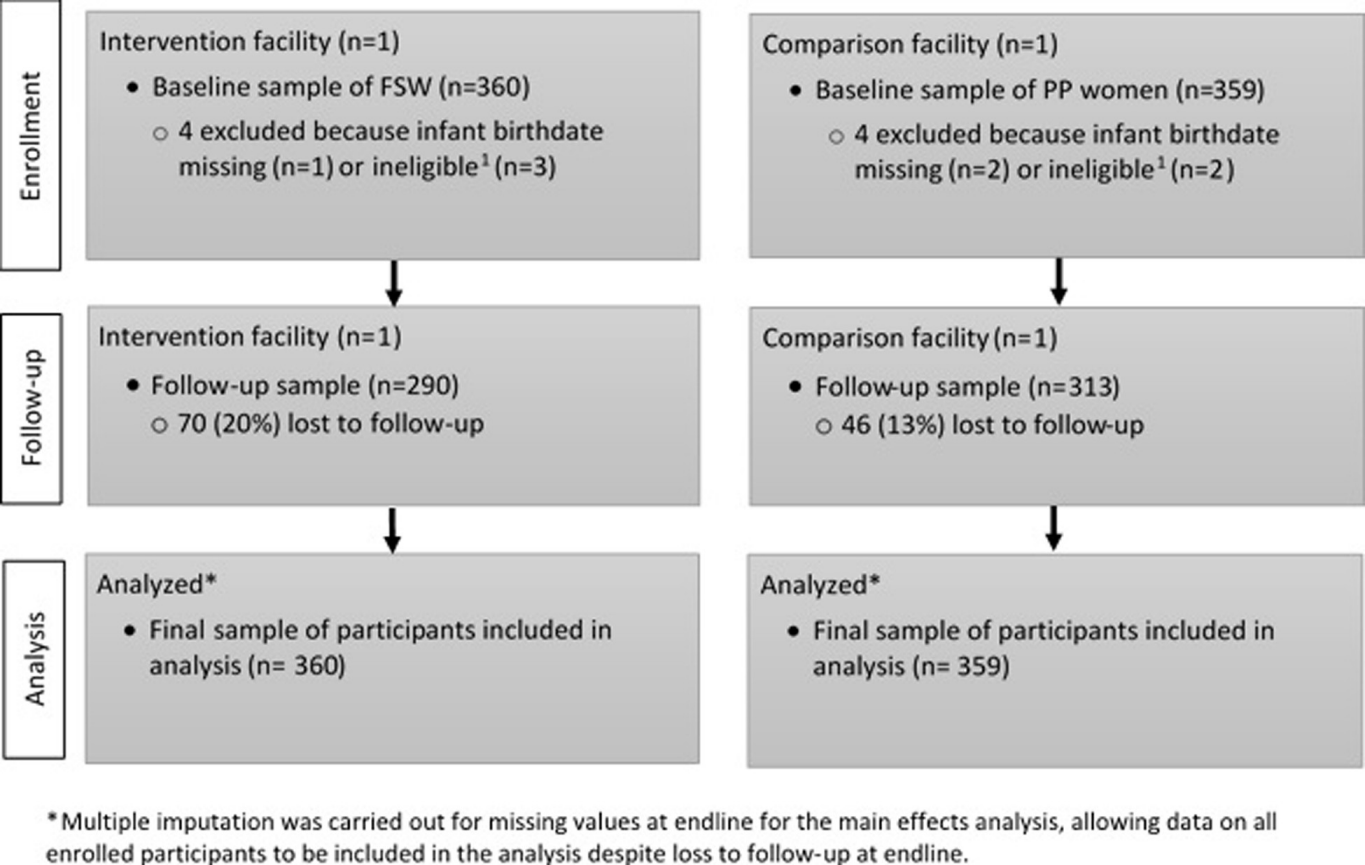
Consort flow diagram.

We enrolled 719 FSW in the study: 360 in the intervention group and 359 in the comparison group (Fig 1). Follow-up interviews were completed with 290 (80%) women in the intervention group and 313 (87%) in the comparison group. The flow of participant recruitment and follow up can be found in Fig 1.

At baseline, study groups differed significantly on a number of characteristics. Intervention group participants were slightly older, less educated and less likely to have never married compared to comparison group participants (Table 1). Intervention group participants first entered sex work slightly later in age. Over half of women in both groups reported having only paying sex partners in the prior 30 days at baseline; 35.3% of intervention participants and 42.9% of comparison participants reported one or more non-paying partners, such as a boyfriend.

**Table 1:** Selected background characteristics of FSWs at baseline by study group. Student t-test used for continuous variables; Chi-square test used for categorical variables (non-imputed data).

	Intervention n = 360	Comparison n = 359	p-value
	(% or mean)	% or mean	
Mean age (range)	29.9 (16–48)	27.8 (17–48)	<0.0001
Highest level of education completed			
Some primary school or less (REF)	41.1	37.8	0.61
Completed primary school	46.4	50.0	
Completed secondary or more	12.5	12.2	
Marital status			
Single, never married (REF)	30.3	46.2	<0.0001
Divorced/separated/widowed/married^1^	69.7	53.8	
Has a primary non-paying sexual partner	39.2	42.5	0.36
Mean age began sex work (range)	24.0 (9–43)	21.5 (5–46)	<0.001
Sexual partner type in prior 30 days			
Paying only (REF)	63.6	56.0	0.17
Both paying and non-paying	35.3	42.9	
Non-paying only or no sex partners	1.2	0.9	
Mean number of paying clients in past 7 days (range)	8.0 (0–35)	8.1 (0–56)	0.96
*Sexual Health*			
Sought STI treatment in prior 6 months	38.6	39.7	0.76
Tested for HIV in prior 6 months^2^	70.3	80.1	0.002
HIV positive^3^	19.4	16.8	0.38
*Reproductive Health*			
Ever been pregnant	95.6	93.9	0.32
Mean number of previous pregnancies (range)	2.7 (0–8)	2.3 (0–9)	0.0002
Mean number of living children (range)	2.1 (0–8)	1.9 (0–8)	0.10
Ever had an abortion	17.5	12.8	0.08
Mean number of abortions among those who have had an abortion (range)	1.4 (1–7)	1.4 (1–7)	0.74
Currently pregnant^3^	4.7	3.1	0.47
Sought FP service in prior 6 months	30.3	80.8	<0.0001

1. Only one person in each study group reported being currently married.

2. Comparison group n = 356

3. n = 351 intervention group and n = 350 for comparison group.

Nearly all women had been pregnant at least once. Fewer comparison group participants (12.8%) reported ever having had an abortion than intervention group participants (17.7%); however, this difference was not statistically significant. Comparison group participants were far more likely than intervention group participants to have sought FP services in the six months prior to the baseline interview (80.8% as compared to 30.3%), as well as STI and HIV services (Table 1).

### Condom use, family planning and unmet contraceptive need

At baseline, all women reported using condoms at least sometimes with their partners; the majority reported consistent condom use with all partners (71.5% intervention, 63.4% comparison) (Table 2). Condom use consistency varied by partner type in both groups. Consistent condom use with paying sex partners in the past 30 days was reported by nearly all women in both groups, but was considerably lower with non-paying partners in both groups.

**Table 2:** Family planning method use and unmet contraceptive need at baseline by study group, among non-pregnant participants. Chi square test use for categorical variables (non-imputed data).

	Intervention n = 344	Comparison n = 346	p-value
	(%)	(%)	
***Condom Use***			
Consistent condom use with all partner types	71.5	63.4	0.02
Consistent Condom Use by Partner Type			
Paying partners	*(n = 335)*	*(n = 343)*	
Consistent condom use with paying partners	89.6	91.6	0.38
Non-paying partners	*(n = 118)*	*(n = 150)*	
Consistent condom use with non-paying partners	36.4	28.0	0.14
***Other FP methods***	(n = 339)	(n = 344)	
Current non-condom modern contraceptive use	47.5	76.3	<0.0001
Specific method use^1^			
Oral contraceptive pills	9.1	26.3	—
Injectable contraceptive	27.1	35.0	—
Implant	5.5	11.8	—
IUD	2.1	0.9	—
Female sterilization	1.5	2.0	—
Other	1.2	0.6	—
No method	53.4	23.7	—
***Dual method use***	30.7	50.5	<0.0001
***Unmet Contraceptive Need***^***2***^	(n = 338)	(n = 346)	
Total unmet contraceptive need	44.1	19.1	<0.0001
Unmet need to space	13.3	8.1	0.03
Unmet need to limit	30.8	11.0	<0.0001

1. No significance testing performed for individual FP methods.

2. Unmet contraceptive need is calculated based on non-condom modern method use only. Those who used only condoms, regardless of consistency, were not included among those with met contraceptive need.

Non-condom, modern method use was more than 60% higher among comparison women at baseline than intervention women. Injectable contraceptives were used most frequently in both groups followed by oral contraceptive pills; few women reported using longer acting or permanent methods, such as implants, IUDs or sterilization.

Baseline unmet contraceptive need among intervention women was 44.1%, but significantly lower in the comparison group (19.1%) with more than half of unmet need in the both groups being unmet need to limit pregnancies.

### Main effects

Controlling for education, age and non-paying partner changes from baseline to endline, we found that the intervention had a significant positive effect on non-condom, modern method use (OR = 1.38, 95%CI (1.04, 1.83)) (Table 3). There was no observed significant effect of the intervention on dual method use, which was most strongly predicted at endline by change in partner type (from both paying and non-paying to paying only), (OR = 4.42, 95%CI (3.31, 5.90)).

**Table 3:** Parameter estimates and exponentiated odds ratios for treatment*time and change in partner type*time effects on primary outcomes^1^, imputed data.^2^

	Treatment group*Time			Change partner type*time		
Outcome	Parameter estimate	std. err	p-value	Parameter estimate	std. err	p-value
Non-condom method use	0.321	0.1444	0.0268	-0.091	0.121	0.4502
*OR (95% CI)*	*1*.*38*	*(1*.*04*, *1*.*83)*		*0*.*91*	*(0*.*72*, *1*.*16)*	
Dual method use	-0.270	0.1723	0.8757	1.485	0.1474	<0.0001
*OR (95% CI)*	*0*.*76*	*(0*.*69*, *1*.*37)*		*4*.*42*	*(3*.*31*, *5*.*90)*	
Consistent condom use	-0.601	0.1594	0.0002	2.730	0.1733	<0.0001
*OR (95% CI)*	*0*.*545*	*(0*.*40*, *0*.*75)*		*15*.*33*	*(10*.*90*, *21*.*55)*	

1. GLMM

2. Models adjust for age and education

Because of the strength of association between partner type change and dual method use we also examined the relationship between change in partner type and consistent condom use. We observed a statistically significant drop in consistent condom use with both partner types in the intervention group compared to the comparison group (OR = 0.55, 95%CI (0.40, 0.75)). However, the strongest relationship was between change in partner type and consistent condom use. Women who had both paying and non-paying partners at baseline, but had only paying partners at endline were significantly more likely to report consistent condom use compared to those who added a non-paying partner or those who maintained either both partner types or only paying partners over time (OR = 15.33, 95%CI (10.90, 21.55)).

### Condom use characteristics

We further explored FSW condom use with both paying and non-paying sex partners (Table 4). Although nearly all participants in both study groups at both time points reported using condoms at last sex and always in the prior 30 days with a paying partner, substantial numbers in both groups reported condom negotiation with paying partners as somewhat or very difficult; more than one-third in both groups at both time points reported few or none of their paying partners were willing to use condoms at each sexual encounter. Across study groups, condom use with non-paying sex partners was less frequent and more difficult to negotiate than with paying partners.

**Table 4:** Condom use characteristics by partner type among respondents who reported having each partner type, baseline and outcome data (non-imputed data).^1^

	Baseline			Outcome^2^		
	Intervention n = 360	Comparison n = 359	p-value	Intervention n = 288	Comparison n = 307	p-value
	(%)	(%)		(%)	(%)	
***Paying partners***						
Had ≥1 paying partner in past 30 days	98.9	99.2	0.71	94.5	92.6	0.34
*Among those with paying partners*	*(n = 356)*	*(n = 356)*		*(n = 272)*	*(n = 284)*	
Used a condom at last sex	98.9	98.6	0.74	98.5	99.7	0.16
Frequency used condoms with paying^3^ sex partners in past 30 days						
Never or Sometimes	0.3	1.7	0.02	4.4	3.2	<0.0001
Most of the time	9.4	5.1		13.6	3.2	
Always	90.3	93.1		82.0	93.7	
Typically, how easy or difficult to get paying partners to use condoms						
Very difficult	12.1	23.9	<0.0001	16.2	15.5	0.0006
Somewhat difficult	31.7	34.8		27.2	41.6	
Somewhat easy	17.7	23.3		17.7	18.3	
Very easy	38.5	18.0		38.9	24.6	
Proportion of paying partners typically willing to use condoms at each sex						
Few or none	33.2	45.8	<0.0001	30.4	47.2	<0.0001
Most	37.1	46.6		32.3	31.5	
All	29.8	7.6		36.3	21.3	
***Non-paying partners***						
Had at least one non-paying partner in past 30 days	35.8	43.7	0.03	49.5	49.2	0.58
*Among those with non-paying partners*	*(n = 128)*	*(n = 157)*		*(n = 142)*	*(n = 150)*	
Used a condom at last sex	46.1	40.8	0.36	45.5	42.0	0.56
Typically, how easy or difficult to get non-paying partners to use condoms						
Very difficult	43.0	54.1	0.09	51.4	43.4	0.25
Somewhat difficult	21.1	10.8		20.4	18.0	
Somewhat easy	10.9	10.8		6.3	7.3	
Very easy	25.0	24.2		21.8	31.3	

1. P-values presented are for Chi Square test comparing variable across treatment groups.

2. At follow-up, 1 woman in the intervention group and 4 in the comparison group reported having no current sexual partners.

3. 4 missing in intervention group (baseline n = 356) and 6 missing in comparison group (baseline n = 353) at baseline for this comparison.

### Intervention acceptability and service uptake

Most participants in both study groups agreed that receiving FP information from the DIC provider and from peer educators was acceptable (Table 5); however, DIC service satisfaction was significantly higher among comparison women than intervention women.

**Table 5:** Service uptake, satisfaction and acceptability at follow-up (non-imputed data). Chi square test for categorical variables.

	Intervention n = 290 (%)	Comparison n = 311 (%)	p-value
In past 6 months received following service at DIC			
HIV counseling and testing	12.0	22.0	0.001
STI	4.8	3.7	0.31
Condoms	12.7	8.6	0.10
FP	15.1	33.2	<0.0001
Level of service satisfaction at DIC			
Not at all satisfied or somewhat unsatisfied	22.0	1.9	<0.0001
Somewhat or very satisfied	78.0	98.1	
Agree that getting FP information at DIC is good	99.0	100.0	0.60
Agree that getting FP information from PEs is good	92.1	92.0	0.97
Agree that they prefer to get FP services at DIC	95.2	78.0	<0.0001
Member of a peer support group	44.8	27.2	<0.0001
	*(n = 130)*	*(n = 85)*	
Attended a peer support group meeting in past 3 months among those who are peer support group members	65.4	35.3	<0.0001

## Discussion

Our findings suggest an enhanced integrated health service intervention can improve uptake of non-condom, modern methods among FSW–an important strategy to reduce unmet contraceptive need, unintended pregnancies and their consequences. As with the general population of adult women in Kenya and elsewhere in the region, FSW reported high reliance on short-acting contraceptive methods despite very high unmet contraceptive need to limit pregnancies. Over reliance on shorter-acting FP methods can create unnecessary barriers to effective contraception among women who desire no additional children [27]. Once contraceptive implants were made available through the DIC, demand outstripped supply. Future programming should include making longer-acting or permanent FP methods readily available and helping women to choose FP methods aligning to their fertility desires.

It is important to note that among long-acting, reversible contraceptive methods, IUDs carry with them additional considerations for use among women with higher than average risk of STIs, which includes many FSW.[28, 29] In this study, and consistent with global guidance and Kenyan national guidelines, women were counselled using an informed choice approach that examined their individual needs, desires and situations. While current guidance calls for caution in the use of IUDs among women who are at elevated risk of STIs, they also recognize that not all women who self-identify as sex workers share the same life situations and STI risks. As such, the IUD remains a viable option for certain women who self-identify as sex workers and should not be categorically eliminated as an option, but rather selected in close consultation with a qualified health provider, taking into consideration the individual woman’s STI risks and other relevant factors. According to the 2015 World Health Organization’s Medical Eligibility Criteria for Contraceptive Use, “Many women with increased risk of STIs can generally undergo either copper-bearing IUD (Cu-IUD) or LNG-IUD initiation (MEC Category 2). Some women at increased risk (very high individual likelihood) of STIs generally should not have an IUD inserted until appropriate testing and treatment occur (MEC Category 3).”[28] Based on Kenyan and international guidance, IUDs were made available as an option for women in this study; however, during the study, no woman initiated an IUD through the DIC; two women in the intervention group started using an IUD that they received at a health facility elsewhere(.

Results also revealed that, although women are aware that condom use is important to prevent STIs, and unintended pregnancies, their control over such use can be limited. Overall, women reported using condoms less consistently with non-paying partners than with paying partners—an important finding noted in other studies [13, 14]. Coupling the sometimes dangerous conditions under which FSW work with commonly described difficulties negotiating consistent condom use, FSW can be vulnerable to adverse SRH outcomes such as unintended pregnancy and STIs[1, 30]. Using non-barrier, modern contraceptive methods (e.g. hormonal contraceptives) with condoms is crucial to curbing unintended pregnancies and their subsequent potential adverse health, social and economic consequences. These results highlight that other strategies to reduce HIV and STIs are needed. Alternative HIV prevention methods such as oral pre-exposure prophylaxis (i.e. PrEP) and microbicides may be critical for members of this population.

This study’s results support previous research indicating self-reported condom use–a main component of our primary outcome–may indeed be problematic [31–33]. Inconsistencies between self-reported condom use, ability to negotiate condom use, and other markers of condom use, such as seeking STI and HIV services, or reporting unintended pregnancies and abortions, indicate that self-reported consistent condom use may be less than optimal measure of actual condom use behavior in this population.

### Limitations

Despite several strengths, a number of study limitations may have affected the observed outcomes. We sampled only FSW attending DIC services in the two study communities, limiting our ability to generalize findings to the larger FSW community. At the time of the study, DICs were new, limiting our access to the population of interest. However, given our large sample of women (n = 729 enrolled at baseline) and the consistency of findings with similar research, women enrolled in the study provide important information relevant to the wider population of FSW in Kenya and elsewhere. In terms of sampling, the migrant nature of the study target population could create substantial LFT; we inflated our estimate by the expected level of LTF to ensure the sufficient size. Although this inflation covered the actual LTF and sensitivity analyses indicate no significant differences between those remaining in the study and those LTF, it is possible that unmeasured differences influenced our findings.

The quasi-experimental study design carries with it risk of selection bias. The choice of a quasi-experimental study design was largely driven by two factors. First, the experimental intervention was a combined community-/health facility-based intervention. Randomizing individuals within a community would have been impractical; the whole community needed to be selected to receive the intervention or not. Second, because large groups of accessible women self-identifying as FSW are limited in Kenya, a cluster-randomized trial would have been impractical; thus, we elected to identify two areas with known FSW populations. We intended to select communities sufficiently separated to prevent contamination, but not so separate that the characteristics of the women and available services were completely dissimilar. Despite these efforts, our baseline analyses indicated the two groups differed substantially on several important characteristics. In anticipation of this, we centered the statistical approach on the DD estimate of the intervention’s impact. Lastly, by including only one DIC in each group, due to the limited number available, factors such as provider personality and work-related attributes of the DIC staff could have substantially impacted the delivery and uptake of the intervention. Baseline differences between the two groups in terms of FP method uptake and service satisfaction at follow-up indicate that these factors may have been important. Lastly, because non-condom method use was lower in the intervention group at baseline, increases in use would be easier to achieve than in the comparison group where non-condom method use was high in the comparison group at baseline.

## Conclusion

This study contributes to the relatively small body of evidence addressing the broader SRH needs of FSW. Integrated services providing convenient access to family planning, HIV counseling and testing, and screening, diagnosis and treatment of other STIs may better address the SRH needs of FSW. Interventions targeting FSW without addressing the knowledge, attitudes and behaviors of their male sexual partners will likely continue to have only limited impact. More research is needed to identify strategies to engage men to change their behaviors and reduce the risk of unintended pregnancy, and the transmission of HIV/STIs.

## Supporting information

S1 Checklist Checklist 1. Trend checklist for the Kenya FSW study.
(PDF)

S1 File Study protocol. Final study protocol.
(PDF)
