# Supplementary material for: Addressing broader reproductive health needs of female sex workers through integrated family planning/ HIV prevention services: A non-randomized trial of a health-services intervention designed to improve uptake of family planning services in Kenya
Source: PLoS One. 2019 Jul 24;14(7):e0219813. doi: 10.1371/journal.pone.0219813 (PMC6655688; doi:10.1371/journal.pone.0219813)
Supplement: S1 File — Final study protocol. (PDF) [file pone.0219813.s002.pdf]

Development and Evaluation of an Intervention to Increase Family Planning Use among Female Sex Workers in Kenya

Protocol Version 2.0
PHSC Study #10285

Date: 12 February 2013

Funded by: United States Agency for International Development through the Prevention Technologies Agreement Cooperative Agreement: #GHO-A-00-09-00016-00

Sponsor: FHI360
Chancery Blvd., 2nd Floor
Valley Road, Nairobi, Kenya
Telephone: 254-020-282-4000

Sites: Rift Valley Province, Kenya

Principal Investigators:

FHI360:
Lisa Dulli, PhD, MHS
Chancery Blvd., 2nd Floor
Valley Road, Nairobi, Kenya
Telephone: 254-20-271-3913 x55310
ldulli@fhi.org

Amy Corneli, PhD, MPH
P.O. Box 13950
Research Triangle Park, NC 27709 USA
Telephone: 1-919-544-7040 ext. 11248
acorneli@fhi.org

Rose Otieno-Masaba,
Chancery Blvd., 2nd Floor
Valley Road, Nairobi, Kenya
Telephone: 254-20-271-3913
rmasaba@fhi.org

GoldStar Kenya:

John Ndiritu
Nakuru, Kenya
Telephone: 254-051-2215568
JNdiritu@fhi.org

Study Summary

Title: Development and Evaluation of an Intervention to Increase Family Planning Use among Female Sex Workers in Kenya

Study #: 10285

Purpose: To develop and evaluate of an intervention designed to improve consistent contraceptive use and dual method use in particular, among female sex workers (FSWs) in Rift Valley Province in Kenya.

Design: A qualitative descriptive study will be conducted to develop the intervention. A quasi-experimental, non-randomized, two-group, pre-/post-intervention design will be used to evaluate the intervention.

Study Population: FSWs living in Naivasha, Gilgil, and Nanyuki, Rift Valley Province.

Study Duration: Phase I - 3 months

Phase II - 12 months

Objectives: Develop an intervention designed to increase contraceptive use among FSWs by incorporating formative data and behavioral theory and by involving the target audience and service providers in the development of the intervention.

Examine the effectiveness of the intervention at increasing non-barrier modern contraceptive use and dual method use among FSWs.

Assess the feasibility and acceptability of the intervention, on the parts of both the clients (FSW) and service providers.

Estimate the unit cost to provide these services to one client.

Study Site: Rift Valley Province, Kenya

Acronyms and Abbreviations

APHIA	AIDS, Population and Health Integrated Assistance
DIC	Drop-in center
DRH	Division of Reproductive Health
FAIR	Family AIDS Initiative Response
FHOK	Family Health Options Kenya
FP	Family planning
FGD	Focus group discussion
FSW	Female sex worker
GSKenya	Gold Star Kenya
HTC	HIV testing and counseling
HIV	Human Immunodeficiency Virus
KNASP	Kenya National AIDS Strategic Plan
MARPS	Most at-risk populations
MoPHS	Ministry of Public Health and Sanitation
MMS	Ministry of Medical Services
NASCOP	National AIDS and STI Control Program
RH	Reproductive health
SRH	Sexual and reproductive health
STI	Sexually transmitted infection
USAID	United States Agency for International Development

Table of Contents

Study Summary....2
Acronyms and Abbreviations 3
Table of Contents 4
Introduction....5
  Background....5
  Interventions Targeting FSW 5
  Kenya 6
  Intervening to Improve Contraceptive Use and Dual Protection 7
Goal and Objectives....8
  Study goal 8
  Objectives 8
Methods 8
  Study Setting....8
  Study Design 9
  Phase I: Formative Research and Intervention Design 10
  Phase II: Intervention Evaluation.... 13
  Sampling and Sample Size Estimate 14
  Data Collection 15
  Measures 16
  Data Analysis and Hypotheses.... 18
  Instrument Development and Pretesting.... 18
  Data Management.... 18
Ethical Considerations 18
  Informed Consent.... 18
  Description of Risks and Benefits 19
  Protection of Privacy and Confidentiality.... 20
  Reimbursement 20
  Training on Human Subjects Projection 21
Study Implications 21
References.... 22

Introduction

Background

Although considerable public health efforts have targeted female sex workers (FSW) over the past decade, the majority of research and interventions carried out among these women have focused largely on the prevention and treatment of human immunodeficiency virus (HIV) and other sexually transmitted infections (STI). Far less attention has been paid to other sexual and reproductive health (RH) needs of women who engage in the exchange of sex for money or goods (transactional sex); however, the factors that place these women at elevated risk of acquiring HIV and other STIs are the same factors that can place them at risk for other RH health problems, such as unintended pregnancy. Multiple sexual partners, coupled with the low or inconsistent use of barrier protection (e.g. male or female condoms), can place women who engage in transactional sex at high risk for a variety of adverse sexual and RH outcomes.

Only a handful of studies has examined a wider range of sexual and reproductive health issues among FSWs beyond STI and/or HIV. These studies have looked at issues such as induced abortions, unintended pregnancies and contraceptive use, among others. Induced abortion is a common issue among sex workers reported in many countries [1-5]. Unmet contraceptive need is also an important problem with a number of studies reporting modern contraceptive method use among FSW to be quite low, with the exception of condoms [1,5-8]. Among those FSW who do use modern contraceptive methods, inconsistent use and frequent method switching are frequently reported [4,5].

Condom use has been a major focus of most HIV and STI prevention strategies targeting FSWs. These efforts appear to have been relatively successful with the majority FSW in many of the studies reviewed, reporting very high rates of condom use [1,3,6,7,9,10]. Promoting barrier protection, such as male and female condoms, is one strategy to also address contraceptive need because of the dual protection effect of condoms against both the transmission of STIs, including HIV, and pregnancy. However, many of these same studies indicate that, although self-reported condom use is high, they are often used inconsistently and findings from some studies indicate incongruities in the self-reported condom use behavior of FSW [4,6,10]. For example, women who report frequent condom use, also report difficulty negotiating condom use with paying partners, and being paid higher to engage in sex without condoms than with condoms. In a study conducted in Mombasa, Kenya, men who paid for sex said they did so for pleasure and that they did not find sex with a condom pleasurable [11]. Additionally, research indicates that many FSW who use condoms regularly with paying partners, do not do so with their non-paying or emotional partners. These factors leave FSW at-risk not only for contracting sexually transmitted infections, but also highlight the need for the use of additional contraceptive methods to help prevent unintended pregnancy.

Interventions Targeting FSW

The stigma attached to sex work and the illegal nature of the activity in many countries make identifying and reaching out to these women a challenge. As a result, the mainstay of most prevention programs that target FSWs is typically a combination of peer-led education and health promotion activities coupled with condom distribution. Accessible health services, which most commonly include STI management, HIV counseling and testing, and linkages to HIV treatment for HIV positive individuals, also play a major role in many prevention programs [12-20].

As previously noted, the majority of these interventions have aimed to reduce HIV transmission in this population through promoting safer sexual practices, including correct and consistent condom use. Less commonly, interventions have targeted the reproductive health needs of FSW through post-abortion care services and by integrating family planning education into HIV prevention peer education. Given FSW's elevated risk for unintended pregnancies – in addition to acquiring sexually transmitted infections, including HIV -- programs targeting FSW with comprehensive RH services are needed.

Kenya

The 2009-2013 Kenya National HIV/AIDS Strategic Plan (KNASP III) calls for targeted interventions for most at-risk populations (MARPs), including sex workers (SWs), to effectively reduce the incidence and burden of HIV in the country [21]. Further, Kenya's National Guidelines addressing HIV/STI prevention among sex workers emphasized the need and call for programs to address not only the promotion and distribution of male and female condoms, but also access to reproductive health services including family planning, post-abortion services, cervical cancer screening, emergency contraception, and post HIV exposure prophylaxis.

In 2008, FHI conducted a descriptive study of FSWs in Coast and Rift Valley Provinces in Kenya to generate a better understanding of the contraceptive health needs of FSWs in these regions [22]. The study involved interviews with 300 FSW in Mombasa (Coast Province) and 297 FSW in Naivasha (Rift Valley Province). Additionally, 4 focus group discussions (FGD) were held in each of the two sites with a subset of the women who participated in the interviews.

Survey findings revealed unmet reproductive health needs among the sample [22]. When reporting current contraceptive use, 38% reported dual method use (condoms plus another modern method) and 16% reported using a non-condom modern method alone. In addition, 39% reported using condoms as the only form of contraception. Relying on condoms as the main source of contraception may not be an effective contraceptive choice given the inconsistent condom use also reported in the survey. For example, 90% of women reported using a condom at last sex with a paying partner yet 46% said negotiating condom use with clients is "difficult" or "impossible." Although the majority of women reported using condoms at last sex with a paying client, only 41% of respondents reported using a condom at last sex with an "emotional" partner. FGD participants reinforced the survey findings by acknowledging that even though condoms are an ideal method for FSW to use as they provide dual protection from pregnancy and STIs/HIV, they are also difficult to use in practice because partner negotiation is difficult, tearing and breakage occurs, and FSWs may forget to use while drunk or high.

Survey respondents also described their fertility desires. Sixty percent reported they did not want any more children and 16% did not want children in the next two years. Further, 52% of respondents reported ever having an unintended pregnancy and 37 % reported ever having an induced abortion, accounts which are consistent with findings from other studies that have examined reproductive and sexual health needs among beyond HIV and STI prevention [22]. FGD participants also described that FSWs in their area experienced high rates of unintended pregnancy and elective abortions even with the frequent use of condoms and other modern contraceptives. Participants said FSWs knew well the array of consequences associated with unintended pregnancy, such as loss of work and clients and the possibility of domestic violence and financial hardship. Respondents described the difficulty faced by FSWs of weighing the consequences of abortion versus having a baby for which they are not prepared. While oral contraceptive pills were described as the main hormonal contraceptive method among FSW because they can be obtained easily, FGD participants said FSW are very much interested in other methods that require less effort on their part, such as injectables and implants; little support was mentioned for IUDs.

In the context of a program targeting HIV prevention among sex workers in Nakuru, Kenya, FHI also conducted one FGD as part of a quality improvement activity to discuss needs and barriers among FSWs to accessing family planning services [23]. The focus of this FGD was to explore perceptions of services provided at drop-in centers (DIC) that are sites where a variety of services are made available for FSWs. DICs are staffed by volunteers, and are usually opened for few hours during the day. They provide condoms, HIV/AIDS information and referrals to HIV testing sites or health facilities. They also provide information on family planning (FP) and referrals to health services for those interested in learning more or obtaining an FP method.

During the FGD, the 13 FSW who participated noted a number of issues that they felt would make services more accessible to FSWs. These included:

- More convenient opening hours for DICs, including late in day to midnight, because FSWs often sleep or take care of other obligations during the day.

- Having FP and HIV counseling and testing (HCT) services offered in the DIC instead of being referred elsewhere
- Services need to be "affordable" or free-of charge

Under the United States Agency for International Development (USAID) funded AIDS, Population and Health Integrated Assistance (APHIA) II program (2005-2010), a number of efforts to reach FSWs with HIV and STI prevention activities took place. Under this project in Rift Valley Province, organizations such as Family Health Options Kenya (FHOK), Family AIDS Initiative Response (FAIR), and Life Bloom Services were working with FSWs to address their health-related needs. FHOK is a health service provision organization and provides a number of health care services that target FSWs including HIV counseling and testing (HCT), family planning, antenatal care, post-natal care, syndromic management of sexually transmitted infections (STI) and HIV care and treatment. These services were offered daily at one established health care facility located near Nakuru, and one to two times per month through outreach/mobile services. In addition to services for FSWs, FHOK also targeted their services to the children and partners of FSWs. FAIR, which collaborates with FHOK, is a community-focused program that targets FSWs with HIV and other prevention services through a peer-education approach. FAIR trained 264 FSW peer educators in the Nakuru area.

The follow-on to APHIA II, APHIAPlus started up in January 2011. Under this new bilateral agreement, many of the previous activities targeting FSWs are being transitioned to a new implementing partner, GoldStar Kenya (GSKenya). GSKenya is planning to develop and implement a comprehensive set of services delivered through drop-in centers in Naivasha/Gilgil and in Nanyuki over the course of the next 1-2 years.

Intervening to Improve Contraceptive Use and Dual Protection

Developing interventions to address the health needs of FSWs can be difficult. As previously noted, FSW are often difficult to identify and reach because of the stigma attached to sex work, as well as the illegal nature of the activities. FSW have reported that they feel discriminated against by providers and are hesitant to seek health care in some health facilities. Also, as noted in the FGD conducted in Nakuru, women who engage in sex work often work during the evening and night, sleeping during the day, when services are typically offered.

FSW are also not a homogeneous group. Women who engage in transactional sex vary. Some women work out of brothels, bars, hotels or on the street and can be more easily reached compared to those who transact sex work from their home. FSW are often highly mobile, moving to where the demand might be - across town or even across the country. In light of these challenges, it is critical that the target group be engaged in the design of any intervention in order to best meet their needs and overcome some of the challenges their work imposes.

Few theory-based interventions for contraception have been developed and rigorously evaluated. In a recent review of the literature, 14 randomized controlled trials that included a theoretical basis were identified, although interventions among high-risk groups, such as female sex workers, were not included in the review [24]. Theories or models most commonly included were the Social Cognitive Theory and the Information-Motivation-Behavioral Skills model [25]. One of the investigators on this protocol (L. Dulli) is currently evaluating the effect of a theory-based intervention to improve access to and uptake of postpartum family planning services through enhanced family planning in immunization services in Rwanda, based on the Health Belief Model. Theories or models are commonly used to develop behavior change interventions as a theory or model specifies a systematic set of relationships to permit one to identify key factors to leverage so the intervention is more likely to have the desired effect. When theory also guides the evaluation, it permits one to better understand why an intervention is or is not successful.

As part of this research, we will identify the theory or model to guide the development and evaluation of the intervention, based on the formative data collected during Phase I, findings from the 2008 descriptive study, and other work. The theory-based intervention will be implemented by GSKenya, and intended to increase consistent contraceptive use and to promote dual method use among FSW in Kenya. GSKenya will implement intervention activities through funding under APHIAPlus, and the formative research and intervention evaluation will be funded by USAID/Washington through the Prevention Technologies Agreement (PTA).

Goal and Objectives

Study goal

The goal of this study is to develop and evaluate a theory-based intervention that could be incorporated into routine health services to improve the sexual and reproductive health of women who engage in sex work if demonstrated to be effective. To accomplish this goal, the study will be conducted in two phases. During the first phase of the study, FSWs and service providers in the study sites will assist in the design of the intervention, in an effort to increase the likelihood that it will be accepted, feasible, and effective. Brief formative research will be conducted with FSWs and service providers, followed by working group meetings with self-selected FSWs, service providers, and GSKenya representatives who will review data and work with study investigators and/or their designees to design the intervention. During the second phase of this study, the intervention will be implemented and evaluated.

Objectives

Objectives of this study are divided by the two phases.

Phase 1 Objective:

The primary objective of Phase I is to develop an intervention designed to increase contraceptive use among FSWs by incorporating formative data and behavioral theory, and by involving the target audience and service providers in the development of the intervention. Formative data includes data collected in Phase 1 of this study as well as data from the 2008 descriptive study described earlier [22].

Phase 2 Objectives:

The primary objectives of Phase II are to:

1. Examine the effectiveness of the intervention at increasing dual contraceptive method use among FSWs.
2. Assess the feasibility and acceptability of the intervention, on the parts of both the clients (FSW) and service providers.
3. Estimate the unit cost to provide these services to one client.

Methods

Study Setting

The study is planned for two sites within Rift Valley Province, Kenya. The first "site" is comprised of two adjacent towns, Naivasha and Gilgil. These two towns are combined into one site because of their proximity, overlap of other health-related services and high level of FSW mobility between the two towns. The town of Nanyuki will serve as the second site. A more detailed description of these towns is found below.

Naivasha town

Naivasha town is located on the shores of Lake Naivasha and along the Nairobi - Nakuru highway. It is host to a high number of migrant workers who have relocated to the town to work in the floriculture, hotel and tourism industry. There are two major truck stops in Naivasha where trucks destined for western Kenya and other Eastern and Central African countries stop. As a result, Naivasha is home to a large population of sex workers.

FSWs' HIV prevention interventions, implemented by Life Bloom Services under APHIA II, had been running in the town area since 2004. The main project activities included:

- Peer education and outreach
- Counseling and testing for HIV
- STI screening and treatment

- Condom promotion and distribution
- Strategic Behavior Change Communication material distribution
- Outreach to identified sex workers' clients, including truckers and men in the informal sector.

This DIC and all related services were closed once funding from APHIA II ended. However, new services have recently (March 2012) been initiated under APHIAPlus, in line with the newly released guidance from the MOH on the basic package of services to FSW.

Service currently being provided to FSW in Naivasha include peer education and health services delivered through a drop-in center staff by a counselor and a nurse. For peer education, there are six modules that the peer educators walk all peers through with sessions being held once a week. The modules are delivered in twelve 45 – 60 minute sessions.

The modules cover the following areas:

- HIV and AIDS; Basic facts, transmission and prevention
- STIs; Basic facts, transmission and prevention
- Correct and consistent condom use
- All you need to know about HIV testing
- FP; methods, advantages of modern contraceptives etc
- Alcohol and other drugs
- Sexual and gender-based violence (SGBV)

In addition to facilitating group sessions among FSWs, peer educators also mobilize their peers to access services through the DIC or through the monthly integrated outreaches. There is a newly established drop-in center that provides HIV testing, STI screening and treatment, RH/FP services, TB screening among other services.

Naivasha town currently has 16 active peer educators. Between May and August 2012, the DIC enrolled 403 FSW. There are approximately 90 new FSW and 100 repeat visits each month. In addition to peer education and health services provided through the DIC, there are adult literacy classes and crafts classes in which 126 FSW are currently participating.

Nanyuki town

Nanyuki town is located on the slopes of Mt. Kenya in Laikipia County approximately 250 km North West of Nairobi. The main economic activity in Nanyuki is agriculture with large commercial wheat farms and floriculture farms as the primary employers. The town also hosts a Kenya Air Force barrack and the British Army maintains a training base in the town. The town is also a popular tourist destination providing a base for mountaineers and other tourists to the Mt. Kenya and Meru National Parks. The presence of a high number of migrant workers who provide labor in the farms, hotels and the presence of military have attracted a high number of sex workers in the town.

Nanyuki currently has a team of 20 trained and active FSW peer educators. As of October 2012, there were 353 FSW enrolled in the DIC, which receives an average of 70 new FSWs and approximately 80 repeat visits every month who come in for different services.

The peer education groups are very fluid since they last only for the period it takes to cover the six modules. However, the FSW have been supported to form self-help and support groups. Nanyuki has four groups registered with a membership of 168 FSW while Naivasha has three groups with a membership of 89 FSW. In addition to health promotion activities, these groups are engaged in different economic and social activities.

Study Design

This study will be carried out in two phases. The first phase of the study will include formative research, using a qualitative descriptive study design, and working group meetings to inform and develop the intervention that will subsequently be evaluated by the study. The purpose of this first phase is to increase the likelihood of

ownership and success of the intervention by engaging the target population from the beginning of the project, including in the design of the services to be offered to them. The second phase of the study will involve the formal evaluation of the effectiveness, feasibility, and acceptability of the intervention.

Phase I: Formative Research and Intervention Design

A step-wise approach will be used, starting with formative research using FGDs with FSWs and service providers. Data will be rapidly analyzed and prepared for subsequent working group meetings with FSWs, service providers, and program planners, including staff from GSKenya, to develop a theory-based intervention to increase contraceptive use, including the identification of specific service delivery components.

Sampling and Sample Size

In the first site, Naivasha and Gilgil, up to four FGDs will be conducted with each of the following groups 1) FSWs who have previously received services from Life Bloom Services, 2) FSWs who have not previously received services from Life Bloom Services, and 3) FSWs who served as peer educators under APHIA II. Approximately 8-12 women will take part in each FGD, although fewer or more women may participate. We will also conduct up to two FGDs with service providers who have provided services targeting FSW through local public and private sector services, including Life Bloom Services. Approximately 6-8 service providers will participate in each FGD, although fewer or more providers may participate depending on the total number of providers who are interested and available.

In the second site, Nanyuki, up to six FGDs will be conducted with FSWs. Approximately 8-12 women will take part in each FGD, although fewer or more women may participate. We will also conduct up to two FGDs with service providers from local health facilities. Approximately 6-8 service providers will participate in each FGD, although fewer or more providers may participate depending on the total number of providers who are interested and available.

Eligibility Criteria

A FSW is eligible to participate in the study if she –

- Is a woman who is sexually active and reports receiving money or goods in exchange for sex in the last 6 months as part of her source of income/livelihood.
- Is between the ages of 16[1] and 49 years.
- Provides oral informed consent.

A service provider is eligible to participate in the study if she/he has provided some type of medical care, contraceptive provision, counseling, or education to FSW in the past year – or will be involved in the future provision of services for FSW -- and provides her/his oral informed consent. For the purposes of this study, the term "service provider" is used broadly and includes clinic personnel who provide medical care and/or contraceptives to FSWs but also counselors and peer educators.

---

[1] This study includes mature minors aged 16 and 17 years old in the sample because they represent an important subset of the sex worker population. In Kenya, the legal age of consent for sexual intercourse is 16 years old (Laws of Kenya, 2006). Furthermore, according to the Ministry of Health's National Guidelines for Voluntary Counseling and Testing (NASCOP, 2001), child sex workers, particularly those over age 15, are considered "mature minors" and may provide informed consent. Because the 16-17 year old participants in our study are sex workers, these minors are legally able to give consent and do not require the knowledge and participation of their parent or guardian. It is not the aim of this study to encourage mature minor's engagement in sex work. Instead, because this study seeks to find ways to increase contraceptive use among all sex workers, regardless of age or socioeconomic level, it is critical to include representatives from the entire spectrum of the sex worker population, including mature minors. FGD facilitators will be trained on how to appropriately respond to distressed mature minors and where to refer appropriate services when requested.

Recruitment

Participants will be selected using purposeful sampling techniques, including a snowballing technique. In Naivasha and Gilgil, peer educators, who were previously involved in APHIA II programs or another health program, will invite FSWs who they know have previously obtained services or received education from Life Bloom Services to participate in a FGD. They will also invite FSWs who they believe have not received services or education from Life Bloom Services to participate in the FGDs. Community mobilizers and/or project staff may also directly visit places where FSWs can be found and invite women to participate in the study. In Nanyuki, peer educators will recruit FSWs, if available during the formative component of the study. If peer educators are not available, project staff will recruit FSWs directly by visiting places were FSWs can be found. Peer educators and staff will encourage FSWs to identify other FSWs they know who may be interested in sharing their thoughts on how to improve contraceptive services for FSWs in the area. Basic eligibility criteria information will be asked such as age, if engaged in sex work in the past 6 months and type of sex work. Recruitment vouchers may also be used. Vouchers would not include the name of the study but rather indicate that the individual is invited to take part in a discussion. It will include information such as the date, time, location of the discussion, and contact information for study staff.

At the end of the FGD, the moderator will provide an overview of the subsequent working group meeting(s) and will ask participants to inform her if they are interested in possibly being a part of the working group meeting. FSWs who are interested will be asked to provide contact information at this time (e.g. name, address, telephone number). Participants will be told that study staff will protect their contact information to the best of their ability. For example, information will be stored in a locked drawer and cabinet. Study staff will subsequently follow-up with interested FSW to coordinate the working group meetings. Staff and peer educators may also mention the working group meetings to FSWs during recruitment for the FGDs. Therefore, FSWs can also participate in the working group meetings without previous participation in the FGDs (in situations where a woman is interested in playing a role in the development of the intervention but is unable to make the FGD, for example).

Service providers will primarily be recruited through the assistance of the local organizations and the investigators on the study. Representatives of the organizations and study investigators will identify staff who may be willing to provide useful information on how to improve contraceptive uptake and continued use among FSWs. Either study investigators or a representative of the organization will then contact individual service providers to inform them of the study and to assess their interest in participating in the FGDs and subsequent working group meeting. Study staff may also directly contact service providers in situations where a service provider is already known from other projects carried out with the organization, for example. During the FGD and also through other mechanisms, such as staff meetings, service providers will be informed of the working group meetings and interested assessed. Contact information (e.g. name and telephone number) will be collected from interested service providers.

Data Collection

FGDs will be conducted by trained moderators in a private location and in the appropriate local language. Each discussion may last up to 2 to 2 ½ hours and will be audio-taped. In addition to being stratified by receiving or not receiving Life Bloom services (for Naivasha and Gilgil), FSWs may be grouped into FGDs by similar characteristics, such as age (e.g. under 25 years old and 25 years and older) or type of work (e.g. home based versus out of home or part-time versus full-time) to maximize comfort and enhance participation. Similarly, service providers may also be grouped by role or organization. Demographic questions will be asked of each FSW and service provider before the FGDs and working group meeting. Demographic questions will be asked in a private setting directly after informed consent is obtained.

At the end of the FGD, FSWs will receive information about readily-available resources for family planning and VCT services.

Research Topics

The 2008 descriptive study gathered data on FSWs' fertility desires and contraceptive preferences. Those data will be used to inform the intervention. Less information was available on service delivery preferences and strategies to promote consistent use of preferred contraceptives. Hence, the FGDs with FSW will:

- Gather information on the barriers and facilitators to obtaining services through programs previously offered through APHIA II, specifically focusing on gender-based norms and inequalities that may serve as barriers to accessing services and using contraceptives.
- Determine preferences and perceived facilitators and barriers to receiving services through several service delivery options such as a health care facility, mobile services, a drop-in center, or peer educators – and to identify solutions for overcoming potential barriers identified
- Explore perceived barriers to using long-acting hormonal conception and dual methods, including management of side effects and consistent use over time
- Explore the salience of key variables in selected behavior-change theories such as self-efficacy and subjective norms in order to determine which behavior change theory might best inform the intervention under development
- Explore message content and strategies for communicating messages that encourage long-acting hormonal conception and dual contraceptive use among FSW

The FGDs with service providers will identify service delivery gaps and suggestions for improvement as well as provide context to delivering interventions to FSWs.

During the working group meetings with self-selected FSWs, service providers, representatives from GSKenya, and study investigators, the formative research data and behavior-change theories will be reviewed. Members of the group will use these data and information about behavior-change theory, as well as their experiences, to design a theory-based intervention to increase contraceptive use. The proposed intervention will also include service delivery components such as the types, number, locations and times of the services to be offered by GSKenya.

Data Management and Analysis

Data will be managed and analyzed by FHI staff (country office and headquarters). Qualitative thematic analysis methods will be used for analysis. Data analysis will begin during data collection by transcribing and translating each audio-taped FGD. When possible, FGDs will be simultaneously transcribed and translated from the original language to English. All transcripts will be typed into a word processing program and password-protected; some transcripts will be handwritten first. Demographic data will be entered into a password-protected database. Transcripts and the database will be stored on password-protected computers. All data will be sent electronically via email to FHI Nairobi and Headquarters or stored directly onto FHI's network. Data management logs may be created track and monitor data collection and transcription.

As soon as possible after the FGDs are conducted, transcript text will be read carefully by the study investigators in order to: (1) ask any questions of the text that may be unclear; (2) point out areas in which interviewing and transcription techniques could be improved; and (3) identify recurrent themes and areas for future probing. As in most qualitative studies, questions may be modified, added to, or deleted from the FGD topic guide during data collection based on information that is learned from conducting and reading the transcripts from the previous focus group discussions. Topic guides will also be modified as appropriate after the training and field-testing. Grounded in qualitative methodology, this approach allows researchers to explore in more detail themes that emerge during data collection, and improves understanding of the overall issues. These modifications to the topic guides will be made after the original approval from the Ethics Committees (EC)/Institutional Review Boards (IRBs) and will not be submitted for approval; however, *only* probes and questions that are related to the overall topics that are described in this protocol and that are similar to the

original questions will be added. Any changes made to the topic guides that are beyond the topics described here will be resubmitted for EC/IRB review and approval prior to use.

Data-derived codes developed through inductive coding and retrieving will be used during analysis. A priori codes for retrieving text for key concepts related to the overall objectives also will be applied to the data. Investigators will determine a coding frame to be used based on the topic guides and the first few FGDs available for analysis. New codes will be added as necessary during transcript analysis. A qualitative data software program, such as QSR NVivo or a similar program, will be used to organize all qualitative data and prepare the data for analysis. Procedures will be put into place to check for inter-coding discrepancies. Once all the transcripts have been coded, textual coding reports will be produced. Data reduction techniques will be used to examine codes in detail for sub-themes and patterns across the FGDs. Summary reports will be developed and recommendations for intervention development will be made.

Practice FGDs

During training, FGD moderators may practice using the FGD guides and demographic questionnaires for FSWs with FSWs from the community. In most cases, individuals who will participate in the training activities will have a prior relationship with the site staff or collaborators, or will be recruited by members of the community who have a prior relationship with staff or collaborators. Individuals will be told that they are only participating in practice activities so that the moderators can improve their moderating skills and the questions can be improved. Also, they will be told that all information gathered during the practice session will be destroyed at the end of the training workshop. They may receive a small stipend for their participant (the same amount provided to research participants for taking part in a FGD). Minor changes may be made to these guides and questionnaires after the practice activities.

Phase II: Intervention Evaluation

The Intervention

Based on findings from the formative phase of this study, and on observations and feedback from providers, a package of enhancements to the current package of services delivered to FSW will be implemented and evaluated.

- FP Messaging for *peer educators* and informational materials material

  Peer Educators are trained using a National Peer Education Manual for Sex Workers. The manual focuses mainly on HIV and STI prevention, Care and Treatment. With the exception of male and female condoms, there is no other information on contraceptive use. For FSW peer educators (PE) to effectively communicate to their peers on family planning, it is important to provide them with accurate information on contraceptive use and the benefits of dual method use. Peer educators will be trained using existing materials to deliver accurate messaging on FP/dual method use, during individual encounters and in group sessions. The focus of these materials will be to provide information that FSW can use to more accurately assess their own perceived susceptibility to an unplanned pregnancy, as well as to promote the benefits of contraceptive use for planning pregnancies. Information on services and facilitated referrals by PE are designed to reduce perceive barriers to FP services.

- Screening for unmet FP need in DICs

  Although the National guidelines for SW Programs include FP as part of the package of services that sex workers should access, the bias towards screening for STIs and HIV testing remains. To enhance uptake of FP among FSWs, it is important to equip service providers with the necessary skills and tools to identify unmet FP needs among their clients. Integration of FP needs screening in the service delivery points will increase the number of FSW accessing FP. This will be done through sensitization of service providers and provision of a brief job aid that can be used to screening clients for unmet need, including unmet need for dual method use.

- Providers FP counseling job aid

  Job aids are important in providing cues to perform an action hence reducing reliance on recall or reference to materials that may not be readily available. The APHIA Plus project will adapt the existing Balanced Counseling Strategy Plus: A Toolkit for Family Planning Service Providers Working in High HIV/STI Prevalence Settings[26] for providers to use within the context of the DICs and in referral sites in the catchment area.

  - Commodities – increasing method mix

  The methods that will be available to all women attending services at the DIC will be expanded. In addition to the current oral contraceptive pills, DMPA, male and female condoms, both the intra-uterine contraceptive devices (IUCD) and implants will be added to the mix of methods available.

  - Hours of DIC operation

  Based on findings from the formative phase of the study, the hours of operation for DICs will be extended into the late evening hours to accommodate the schedules of FSW. Currently services are offered from 8:00 AM to 5:00 PM. However, FSW reported that mornings are not a good time for them to seek services, therefore the operating hours for the DICs will be changed, in consultation with clinic staff, to extend into the evening hours to accommodate the needs FSW clients. The proposed time change is 10:00 AM to 8:00 PM.

The conceptual model of the intervention effects is depicted in Appendix 1.

Study Design

The nature of the target population, which is hidden and somewhat transient, creates some design challenges to overcome in order to conduct a rigorous evaluation of the intervention. Additionally, the intervention will be promoted within an entire community, necessitating that separate communities be chosen to avoid contamination. Because large populations of FSW are confined to a few larger cities in Kenya, it would be impractical to randomize communities to treatment and control groups. To address these challenges, the evaluation phase of the study will entail a quasi-experimental, two-group, pre/post-test study design. Data will be collected in two waves, as indicated in Figure 1.

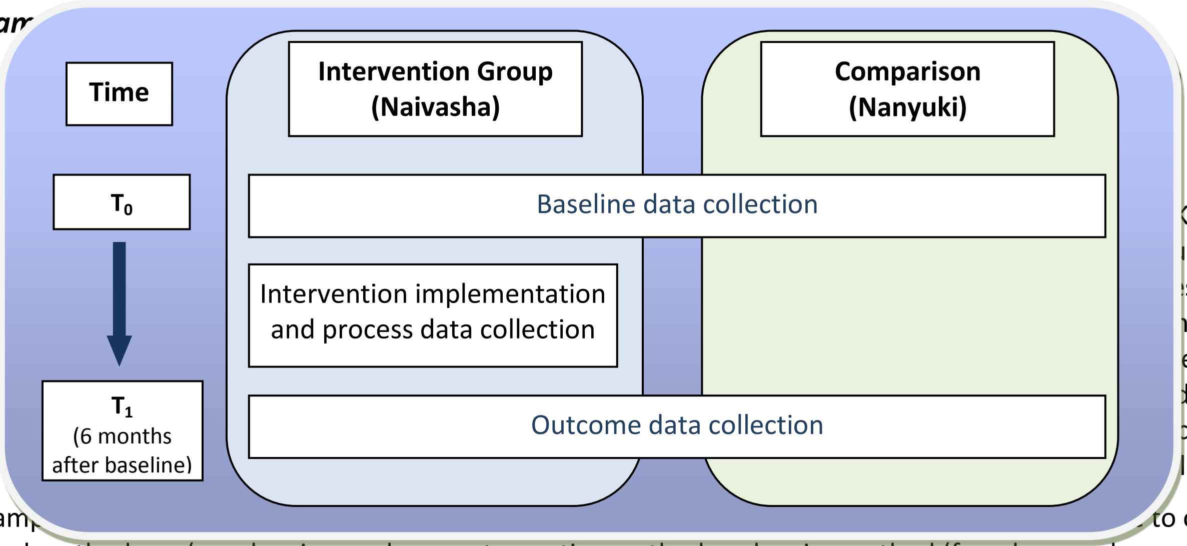

Figure 1. Graphic representation of study design.

Sam[illegible]

Th[illegible] providers w[illegible]

FS[illegible]

R[illegible] Kenya upon w[illegible]ide non-p[illegible]s are used t[illegible]non-p[illegible]ercent of F[illegible] only. In t[illegible]dual m[illegible]ley.[22]

Sam[illegible] to outcome in dual method use (non-barrier modern contraceptive method + a barrier method (female or male condoms)). Calculating the sample size to detect a 15 percentage point increase in dual method use from 38 percent to 53 percent using the 2008 survey data by Sutherland and colleagues [22] would result in a sample of 300 FSW in each of the two study groups, for a two-sided test with an alpha of 0.05 and power of 80%. Because attrition in the sample over the course of the study is likely, an additional 20% will be added to the sample size for a total of 360 women per site.

At the outcome wave of data collection, in addition to structured interviews, in-depth interviews will be conducted with a randomly selected subset of FSW. Sample size for these IDIs is up to 12-15 IDIs per study site, with sampling to end once information saturation is reached.

Peer Educators

All peer educators associated with the APHIAPlus FSW program in the two study sites will be interviewed at both waves of data collection. There are currently 16 PEs in Naivasha and 20 in Nanyuki.

Providers

All providers associated with the APHIAPlus FSW program in the two study sites will be interviewed at both waves of data collection. Currently there is one provider in the DIC in Naivasha and one provider in Nanyuki.

Eligibility and Recruitment

Consistent with Phase I criteria, a FSW is eligible to participate in the study who –

- Self-identifies as a FSW. For the purposes of this study, a FSW is defined as a woman who is sexually active and reports receiving money or goods in exchange for sex in the last 6 months as part of her source of income/livelihood.
- Is between the ages of 16 and 49 years.

FSW will be recruited to participate in the study at the drop-in centers on an ongoing basis until the desired sample size is achieved. Because referrals from peer educators is the primary source of FSW visits to the drop-in centers in both sites, peer educators will also be given information to share during their PE meetings with FSW on the study; however, only those FSW who come to the DIC for services will be enrolled. Peer educators will be informed of the study purpose and description. They will be given printed recruitment materials to distribute to FSW who are interested in participation.

Within the drop-in center, all eligible FSW will be screened by a trained data collector in a private room. Those who choose to participate will be interviewed after informed consent is obtained. Contact information will be collected and the participant notified that we will ask to interview them once again in six (6) months. As noted in the section on ethical considerations, FSW will be given a small allowance for their participation to compensate them for their travel and time. For the follow-up interview, FSW will be contacted by telephone to return. The data collector will try 3 times to contact the participant at weekly intervals. After 3 attempts to contact the participant, a peer educator will be asked to track the participant and request her return. If after one week the peer educator is unable to locate the woman, she will be counted as lost to follow-up.

Peer educators eligible to participate in the study will include all formally trained PEs who are currently registered as PEs with the APHIAPlus project in both intervention and comparison sites at the time of data collection. They will be contacted directly through the APHIAPlus project to be informed about the study.

Providers eligible to be included in the study will be all health care providers and workers who are engaged by the APHIA Plus project in both intervention and comparison sites to provide FP and other health services to FSW as part of the intervention. Providers will also be contacted and informed of the study through the APHIAPlus project.

Data Collection

<u>FSW</u>

Structure interviews

Data will be collected using structured questionnaires administered by trained interviewers during face-to-face interviews. The same questionnaires will be used at both waves of data collection, with the exception that a few

questions on intervention exposure and service satisfaction among those in the intervention arm will be added during the outcome wave.

In-depth interviews

In addition, a 12-15 FSWs will also be purposefully selected to take part in a qualitative, in-depth interview during the second wave of data collection. Data collected through these interviews will explore experiences with service delivery and acceptability of DIC services.

Providers

Individuals charged with providing FP services to FSW DIC clients will also be interviewed using structured questionnaires administered by trained interviewers during face-to-face interviews. The purpose of these interviews will be to collect data on provider training, experience, FP-related knowledge and perspectives with regard to the implementation of the intervention, including perceived facilitators to success and challenges to implementation. Because the level of knowledge required and the intervention components differ between health providers and PEs, two separate instruments will be used to collect data from these two sets of providers.

Process data

Service statistic data will also be collected monthly to monitor trends in service delivery. These data will be extracted from service provision registers. Additionally costing information will be collected on all aspects of implementation through the APHIAPlus program.

Measures

Outcomes of interest:

Main outcome

- **Objective 1**

For **objective 1**, the outcome of interest is ***dual modern contraceptive method use***, defined as use of a non-barrier contraceptive method plus a barrier contraceptive method. This will be measured by self report of current modern contraceptive method use disaggregated by method. For the purposes of this study, the following will be included as non-barrier modern contraceptive methods: oral contraceptive pills (OCP), injectable hormonal contraceptives, contraceptive implants, intra-uterine contraceptive devices, female sterilization, Standard Days method (SDM), and the Lactation Amenorrhea Method (LAM) for post-partum women who meet the LAM criteria. Barrier modern contraceptive methods include male and female condoms. Male sterilization will not be considered in this study because, by definition, FSW have more than one male sexual partner. Dual method use will be defined as use of a non-condom method in the past month plus reported use of a condom (male or female) at last sex with both paying and non-paying partner (if applicable).

Consistency of use for each of the contraceptive methods will be determined through questions that examine when a woman began her current method, when her last renewal of that method was (e.g. resupply of OCPs, re-injection date for depot-medroxyprogesterone or other injectable contraceptive). For OCPs, women will be asked if and how many pills they missed during the last cycle and what they did, if anything, to compensate for missed pills. For condoms, frequency of use in the prior 1 month and at last sex, by partner type, will be examined.

Other outcomes

- **Objective 2**

For **objective 2, *provider and client perspectives related to FP service delivery*** will be explored through a combination of closed- and open-ended questions.

Acceptability on the part of the provider is assessed by the degree to which the provider believes the activities are relevant to his/her work, important for the client, and should be part of his/her responsibilities. ***Feasibility***

of an intervention refers to the fact that all necessary conditions for the activity must be present. These conditions include things such as relevant policies supporting or providing a framework for the activity, as well as the presence of necessary human and material resources allocated for the activity. Providers will be questioned about their perceptions of the feasibility based on these conditions.

Client's acceptability of the intervention is reflected by clients' perceptions of the degree to which the intervention meets a perceived need, as well as the ways in which that need is met. Questions will explore perceived quality of care, convenience of services, affordability and satisfaction.

- **Objective 3**

-Objective 3 will explore the resources necessary to carry out the intervention by examining the ***unit cost to provide service to one individual.*** This measure will be assessed by calculating the total cost of the pilot intervention and then dividing by the number of FSWs served with FP services. The costs of the intervention will be classified according to three distinct phases: 1) development of the pilot; 2) preparation for service delivery; and 3) service delivery. Costs of activities in the initial development phase for intervention design include labor costs for the project team and consultants, and materials for designing clinical or educational resources and/or a training curriculum. Costs associated with preparing for service delivery include those associated with training providers and/or peer educators and printing of job aids and other materials for the intervention. Costs of service delivery are those associated with implementation of intervention activities via health care facilities, mobile services, and/or peer educators. These will include any incremental costs beyond the existing service delivery costs--such as additional stipends provided to peer educators, additional supervision costs above and beyond the customary, and transport costs for mobile services beyond the customary. To estimate the cost of intervention development, FHI will compile information from labor reports, the financial tracking system, and trip reports. To compute the costs of preparation and service delivery, FHI will work with the implementing partner(s) to identify all of the activities for which cost data will be collected. Finally, the total intervention cost will be divided by the number of FSWs served with the intervention to calculate the unit cost per person.

Other measures:

Data will be collected on the following variables.

<u>FSW</u>

Socio-demographic variables: age, education, other employment (non-sex work), marital status (married, widowed, divorced or never married), residence and residential mobility.

Reproductive health: Data on pregnancy history (# times pregnant, # living children, # elective abortions, # miscarriages) and fertility desires will be collected to inform understanding of contraceptive need in this population.

Family planning: Measures of family planning knowledge and prior use, contraceptive need and reasons for non-use among non-users, will be assessed. Source of contraceptive methods, as well as duration and consistency of use will be examined.

Sexual risk behaviors: Number and types of sexual partners (husband/emotional partner, regular boyfriend, casual (non-paying) partner, paying partner); condom use frequency and consistency by partner type. Self-reported HIV status will also be measured.

***Gender issues*:** Measures that examine condom negotiation ability with both paying and non-paying partners, as well as measures that examine gender-based violence experience in this population will be included.

Other HIV risk factors: Measures of alcohol and intravenous drug use will be collected.

Theory-based cognitive factors: Data on theoretical factors from the Health Belief Model [28] will be collected including: *perceived susceptibility* to and *perceived severity* of an unplanned pregnancy, *perceived benefits* of and *perceived barriers* to family planning.

Process data

In addition to independent variables and outcome measures, the project will collect process data to inform interpretation of study results, including whether and to what extent the intervention is delivered as originally intended. In particular, program data will be collected for: trainings (e.g. # provider trainings held; # of providers trained, by type); service statistics to monitor trends in FP and other service delivery related to the intervention over the course of the study; and data from routine supportive supervisions conducted within the scope of the project. including: # of contraceptive methods distributed, by method; # of new FP clients; # of condoms distributed, by source (peer educator, DIC); # of HIV tests conducted; # of referrals for health services, by service type (FP, HIV, ANC, etc.).

Data Analysis and Hypotheses

A detailed data analysis plan will be developed by the Research Analyst in consultation with the PIs and FHI biostatistician. All analyses will be focused on attaining the stated study objectives.

The first objective will be addressed by comparing the change in dual method use from baseline to the outcome wave of data collection between the intervention and control sites using mixed effect linear models. The first objective will be assessed using a two-sided test at the 0.05 significance level. All analyses will control for age, parity, education.

The main hypothesis to be tested in this study is that the intervention will lead to an increase in dual contraceptive method use among FSW, or:

> FSW in the intervention community will be more likely to report dual method use at outcome than FSW in the comparison community.

Instrument Development and Pretesting

The co-Principal Investigators are responsible for development of the protocol and all study instruments. All questionnaires will be translated into Kiswahili and all interviews will be conducted in either Kiswahili or English according to the participant's choice. Research assistants will be fluent in English and Kiswahili. The Study Coordinator, in collaboration with the PI, will conduct initial instrument pre-testing as necessary, but all RAs will participate in a pre-test exercise during training. The instruments will be revised to improve such features as clarity of the questions, the accuracy of the translation, the completeness of fixed response choices, or the flow of the interview.

Data Management

Data from the second phase of the study will be managed by both Kenya-based and US-based staff. Research assistants trained to conduct interviews will gather data according to the questionnaires developed for this study. Data will be collected using electronic devices (such as Personal Digital Assistants or PDAs). These data will be transferred electronically and stored in FHI 360's database.

All data cleaning, data checks and data analyses will be completed in NC and/or Kenya by the data analyst and/or the TM, in consultation with the FHI 360 biostatistician. Once data are collected, only those individuals directly involved in the study will have access to the electronic data. For the purposes of reports and publication, data will be presented in aggregate form, thereby preventing any deductive disclosure.

After study closure, all data will be archived with FHI 360's data management group.

Ethical Considerations

Informed Consent

For Phase I of the study, oral informed consent will be obtained from each participant before any study specific activities are undertaken. A waiver of *written* documentation of informed consent is requested based on 45 CFR

46.117(c1 and c2). Among participants who are FSW, this request is made because of the sensitivity of the data collected. If names were recorded on the consent form, the written consent document would be the only record linking the FGD participants to the research and the principal risk from these activities would be the potential harm resulting from a breach of confidentiality (45 CFR 46.117(c1). Some but not all participants, however, will provide their contact information if they are interested in participating in the subsequent working group meeting. This information will be stored securely in a locked drawer or file cabinet. Among service providers, the research is of minimal risk and includes procedures where consent is not normally required outside of the research context (i.e. provider feedback on how to improve programs) (45 CFR 46.117(c2). This process if often referred to as “oral informed consent.” Oral informed consent has been used successfully in Kenya in other behavioral studies involving FSWs. It includes all information typically described on written consent forms. The only differences between oral and written consent is the absence of a signature.

Oral consent text will either be read aloud to each potential participant individually or in a group prior to participation depending on the setting. Participants may also read the consent document themselves individually or in a group setting. If information is reviewed in a group, each potential participant will subsequently meet individually with study staff to further review study information as needed, to answer any additional questions, and to obtain consent. Further, prior to joining the group to hear the consent information, potential participants will be asked if they agree to hearing detailed information about the study in a group setting with other FSWs or if they would prefer to hear information first individually. Information will be provided individually for those who request it. Study staff will document that oral consent has been obtained from each participant by providing their signature (study staff) on the oral consent form.

Individual informed consent will also be obtained from individuals who participate in the program planning workshops.

For Phase II of the study, written informed consent will be obtained prior to beginning any study-specific activities. Interviews with both FSW and providers will be conducted in a private area, where the conversation cannot be overheard by others. Informed consent text will be read aloud to each potential participant individually. Participants may also read the consent document themselves individually. If the individual agrees to participate, he/she will be asked to sign or provide his/her mark as appropriate on the informed consent form. Study staff will also document that informed consent has been obtained from each participant by providing their signature (study staff) on the consent form.

Description of Risks and Benefits

There are few anticipated risks associated with participation for FSWs in both phases of the research. Some FSWs may feel uncomfortable or embarrassed when asked questions about contraceptives or their reproductive history. Participants will be told that they do not have to answer any question and they stop participation at any time. Study staff will make every attempt to ensure a comfortable and secure environment in which to interact with participants. Any social harm that occurs, should there be any, will be reported to both KEMRI and FHI360's PHSC per requirements.

There may be no direct benefits to participants from taking part in Phase I or Phase II this research. However, the information provided by participants will be used to inform and evaluate a contraceptive intervention, which may help FSW in the area in the future.

Further, while staff will make every effort to protect individual participants’ privacy and confidentiality, it is possible that participants' involvement in the study (FGDs, working group meetings, evaluation) could become known to others, and that social harms – such as being treated unfairly or discriminated against -- may result (i.e., participants could become known as FSW). All FGD participants will be asked not to reveal the identity of others who participate in the discussion and to not disclose any information discussed within the group to others outside of the group, although this cannot be absolutely guaranteed. Thus a statement regarding this issue will be included in the consent form.

There are no anticipated risks for service providers who participate in the FGDs, working group meetings or evaluation interviews, as they are participating in this study in a professional role.

Protection of Privacy and Confidentiality

All data collection with FSWs will be conducted in private locations. All participants' names will not be written on consent forms or on data collection instruments. Participant first names may be used during the FGDs, if provided by participants, as it facilitates the discussion. However, names will not be written on paper when the discussion is transcribed. Participant ID numbers will be assigned to each FGD participant for organizational purposes. Unique identification numbers will also be assigned for each participant in the evaluation data collection activities.

All participant information will be initially stored securely at the project clinic or study offices at the study sites, and then stored at FHI Nairobi in locked file cabinets in areas with access limited to study staff. All electronic files and databases will be password protected and stored on password-protected computers or servers. Original handwritten transcripts (if FGDs are not directly transcribed into a word processing program), data collection forms, and audiotapes will be stored under lock and key at the study sites. If digital recorders are used, the digital files will be stored on password-protected computers. Handwritten transcripts and audiotapes (or digital files) will be destroyed after data analysis. All data forms and electronic files and databases will be stored securely at the FHI Nairobi office for up to 3 years.

Names and contact information of FSWs and service providers who volunteer to participate in the working group meetings will be recorded for organizational purposes, with their permission. Names will not be included in any formal or informal presentation or documents about the planning process unless permission is provided by the individual. Names of FSW who wish to participate in the working group meeting will be stored in a locked drawer or file cabinet.

For the evaluation phase, identifying information will be collected on participants in order to link their pre- and post-intervention data. No identifying information will be recorded on the questionnaires. Rather, a separate, password protected file will be kept that links participant identifying information to their identification (ID) numbers and their data. This password protected file will be kept on a secure server at FHI 360 and the only persons who will have access to this file are the co-principal investigators. Once data collection and cleaning are complete, this linking file will be destroyed so that only de-identified data remain on file.

Reimbursement

FSWs who take part in the FGDs will be reimbursed 300 Kenya Shillings (KES) (equivalent to US$2.70) to cover the cost of travel and time taken to participate in study activities. Service providers will be provided with a small gift (such as a journal or pen set). Refreshments may be served at the FGDs.

FSW who participate in the program planning workshop(s) will be provided 500 Kenya Shillings to cover the cost of travel and time taken to participate in the workshop(s). Since it is anticipated that the workshop will be at least day-long event, reimbursement takes into consideration time away from paying customers as FSWs often mention to study staff that it is difficult for them to participate in research activities as it takes away time they can be engaged with customers. Service providers who participate in the workshop(s) will receive a small stipend of 500 Kenya Shillings, which is a standard allowance for such workshops.

FSW who participate in the evaluation waves of data collection will be offered a transportation reimbursement for each interview in which they participate because they will be asked to come to a central point for data collection/interview administration. The allowance will be consistent with the allowance in Phase I, 300 Kenya shillings. Therefore if the participant participates in the baseline assessment, she will receive 300 KES transportation allowance; if she participates in outcome data collection, she will receive an additional 300 KES at that time.

Training on Human Subjects Projection

All investigators and study staff will have completed training in human subjects' protection before study implementation begins.

Study Limitations (Evaluation Phase)

Although a comprehensive approach to evaluating this enhanced intervention will be employed, there are limitations inherent to the design of the study. Because of the difficulty that would be faced if trying to randomize individuals or units to treatment and control groups, in terms of both practical and financial difficulties, a quasi-experimental study design has been chosen whereby two separate study sites have been selected: one that will receive the intervention and another that will not.

Selection bias is the greatest threat to internal validity in this design. To address this efforts have been made to ensure the two study sites are as comparable as possible; both sites have established FSW populations with similar existing programming, similar funding sources for programming and of approximately the same size in terms of FSW population have been selected. Data will be collected to allow us to estimate how similar the study groups are at baseline in terms of factors deemed important to the outcomes under study.

Additionally, because of the small number of sites, situational factors could also play an important role in the external validity of the study. For example, if the providers engaged in the activities in the intervention site are more greatly motivated than typical, it could be that the intervention, if shown to be effective, might not work as well in other places without equally motivated providers. Every effort will be made to document perspectives from providers and clients, as well as to monitor and document processes, but these cannot completely rule out situational effects.

Study Implications

This study will fill gaps in existing knowledge regarding effective ways to reach FSWs with comprehensive RH services. While previous research and programmatic work has focused primarily on reducing STI transmission and acquisition among FSWs, this research will identify and evaluate a theory-based intervention and service delivery strategies to reduce unintended pregnancies among FSWs through the use of modern contraceptive methods. Unintended pregnancies can negatively affect the health and well-being of both FSWs and their children. As such, an intervention that reduces unmet need for family planning among this vulnerable population will have numerous positive benefits including reducing rates of induced abortion; reducing rates of violence including domestic violence; improving the economic status of FSWs; preventing mother-to-child transmission of HIV among FSWs who are HIV+; and improving other maternal and child health indicators.

Numerous stakeholders—including FSWs and providers—will be involved at every stage of Phase I and Phase II of the research to increase the likelihood that the intervention will meet the needs of the population and to increase the likelihood that findings are utilized and scaled up. After study results are known, data will be used by GSKenya to enhance their current programs, if needed. Dissemination will include development of research summaries, reports and manuscripts. In addition, dissemination and utilization will include additional stakeholder engagement in planning for scale up. At the conclusion of the study, a technical consultative meeting with key decision-makers from the Ministry of Health, FHOK, FAIR, and GSKenya and other core team partners will be convened. This meeting will provide a platform for joint decision-making and strategic planning for the way forward in Kenya. Further, the implications of this study go beyond Kenya; positive findings from this global priority study can bolster efforts in improving the RH of FSWs worldwide. Efforts to disseminate study data more widely after the conclusion of the project may include technical and program briefs, articles submitted for publication in peer-reviewed journals, conference presentations, and/or regional technical consultations.

References

1. Delvaux T, Crabbe F, Seng S, Laga M. The need for family planning and safe abortion services among women sex workers seeking STI care in Cambodia. Reprod Health Matters. May 2003;11(21):88-95.
2. Delvaux T, Soeur S, Rathavy T, Crabbe F, Buve A. Integration of comprehensive abortion-care services in a Maternal and Child Health clinic in Cambodia. Trop Med Int Health. Aug 2008;13(8):962-969.
3. Elmore-Meegan M, Conroy RM, Agala CB. Sex workers in Kenya, numbers of clients and associated risks: an exploratory survey. Reprod Health Matters. May 2004;12(23):50-57.
4. Feldblum PJ, Nasution MD, Hoke TH, et al. Pregnancy among sex workers participating in a condom intervention trial highlights the need for dual protection. Contraception. Aug 2007;76(2):105-110.
5. Khan MR, Turner AN, Pettifor A, et al. Unmet need for contraception among sex workers in Madagascar. Contraception. Mar 2009;79(3):221-227.
6. Bautista CT, Mejia A, Leal L, Ayala C, Sanchez JL, Montano SM. Prevalence of lifetime abortion and methods of contraception among female sex workers in Bogota, Colombia. Contraception. Mar 2008;77(3):209-213.
7. Todd CS, Alibayeva G, Sanchez JL, Bautista CT, Carr JK, Earhart KC. Utilization of contraception and abortion and its relationship to HIV infection among female sex workers in Tashkent, Uzbekistan. Contraception. Oct 2006;74(4):318-323.
8. Wayal S, Cowan F, Warner P, Copas A, Mabey D, Shahmanesh M. Contraceptive practices, sexual and reproductive health needs of HIV-positive and negative female sex workers in Goa, India. Sex Transm Infect. Oct 27 2010.
9. Thomsen SC, Ombidi W, Toroitich-Ruto C, et al. A prospective study assessing the effects of introducing the female condom in a sex worker population in Mombasa, Kenya. Sex Transm Infect. Oct 2006;82(5):397-402.
10. Tideman RL, Thompson C, Rose B, et al. Cervical human papillomavirus infections in commercial sex workers-risk factors and behaviours. Int J STD AIDS. Dec 2003;14(12):840-847.
11. Thomsen S, Stalker M, Toroitich-Ruto C. Fifty ways to leave your rubber: how men in Mombasa rationalise unsafe sex. Sexually Transmitted Infections. 2004;80:430-434.
12. Feldblum PJ, Hatzell T, Van Damme K, Nasution M, Rasamindrakotroka A, Grey TW. Results of a randomised trial of male condom promotion among Madagascar sex workers. Sex Transm Infect. Apr 2005;81(2):166-173.
13. Kerrigan D, Telles P, Torres H, Overs C, Castle C. Community development and HIV/STI-related vulnerability among female sex workers in Rio de Janeiro, Brazil. Health Educ Res. Feb 2008;23(1):137-145.
14. Luchters S, Chersich MF, Rinyiru A, et al. Impact of five years of peer-mediated interventions on sexual behavior and sexually transmitted infections among female sex workers in Mombasa, Kenya. BMC Public Health. 2008;8:143.
15. Walden VM, Mwangulube K, Makhumula-Nkhoma P. Measuring the impact of a behaviour change intervention for commercial sex workers and their potential clients in Malawi. Health Educ Res. Aug 1999;14(4):545-554.
16. FHI. Sex Workers. [internet]. 2011; http://www.fhi.org/en/Topics/Sex+Workers+topic+page.htm. Accessed 31 January, 2011.

17. Blankenship KM, Burroway R, Reed E. Factors associated with awareness and utilisation of a community mobilisation intervention for female sex workers in Andhra Pradesh, India. Sex Transm Infect. Feb 2010;86 Suppl 1:i69-75.

18. Dandona L, Kumar SG, Kumar GA, Dandona R. Cost-effectiveness of HIV prevention interventions in Andhra Pradesh state of India. BMC Health Serv Res. 2010;10:117.

19. Dandona L, Sisodia P, Kumar SG, et al. HIV prevention programmes for female sex workers in Andhra Pradesh, India: outputs, cost and efficiency. BMC Public Health. 2005;5:98.

20. Dandona R, Dandona L, Kumar GA, et al. HIV testing among female sex workers in Andhra Pradesh, India. AIDS. Nov 18 2005;19(17):2033-2036.

21. NACC. Kenya National AIDS Strategic Plan 2009/10 - 2012/13: Delivering on Universal Access to Services. Nairobi: National AIDS Control Council;2009.

22. Sutherland EG, Alaii J, Tsui S, et al. A mixed-method assessment of the contraceptive needs of female sex workers in Kenya. The European Journal of Contraception and Reproductive Health Care. June 2011 2011;16:173-182.

23. FHI. Quality Improvement Effort to Improve FP Use among Female Sex Workers in Nakuru. Nakuru, Kenya: FHI; 2010.

24. Lopez LM, Tolley EE, Grimes DA, Chen-Mok M. Theory-based interventions for contraception (Review). Cochrane Database of Systematic Reviews. 2011(3).

25. Bandura A. Social Foundations of Thought and Action: A Social Cognitive Theory. Englewood Cliffs, NJ: Prentice Hall; 1986.

26. PopCouncil. The Balanced Counseling Strategy Plus: A Toolkit for Family Planning Service Providers Working in High HIV/STI Prevalence Settings. [internet]. http://www.popcouncil.org/publications/books/2012_BalancedCounselingStrategyPLUS.asp. Accessed January, 2013.

27. Dulli L, Corneli A, Otieno-Masaba R, Kaseje M, Ndiritu J. Development and Evaluation of an Intervention to Increase Family Planning Use among Female Sex Workers in Kenya: Phase I Data. Durham, NC: FHI 360; unpublished data.

28. Strecher VJ, Rosenstock IM. The Health Belief Model. In: Glanz K, Lewis FM, Rimer BK, eds. Health Behavior and Health Education. Second ed. San Francisco: Jossey-Bass, Inc.; 1997:41-59.

Appendices

Appendix 1: Conceptual Model of Intervention Effects

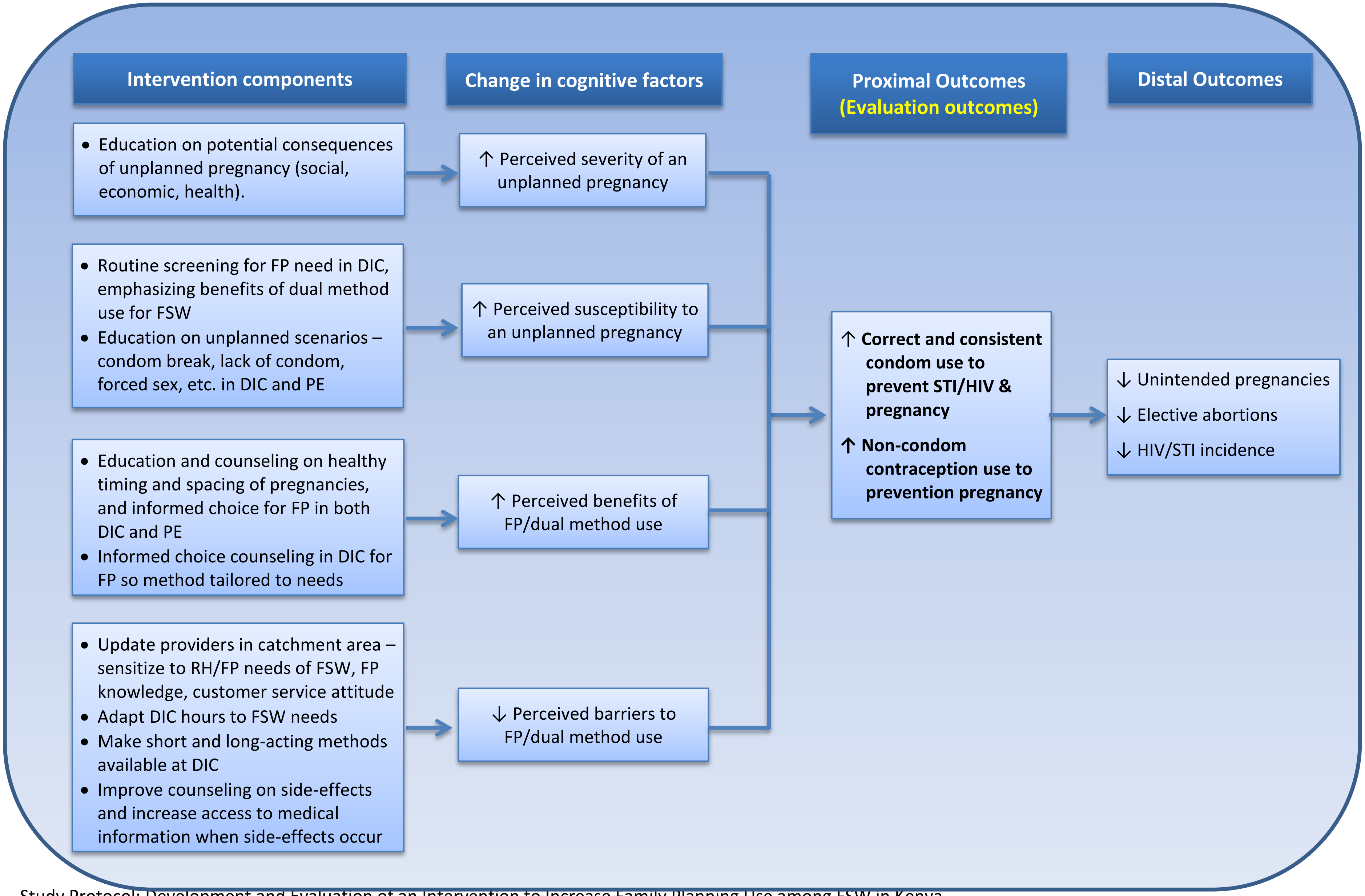

Conceptual model of intervention effects.
